# Clinicopathological Features, Treatment Patterns, and Long‐Term Survival in Patients With Testicular Cancer: A Retrospective Cohort Study

**DOI:** 10.1002/cnr2.70606

**Published:** 2026-06-14

**Authors:** Emanuiela Florentina Rohozneanu, Ciprian Deac, Călin Ioan Căinap

**Affiliations:** ^1^ Department of Oncology The Oncology Institute “Prof. Dr. Ion Chiricută” Cluj‐Napoca, “Iuliu Hatieganu” University of Medicine and Pharmacy Cluj‐Napoca Romania; ^2^ Department of Oncology “Iuliu Hatieganu” University of Medicine and Pharmacy Cluj‐Napoca Romania; ^3^ Anesthesiology and Intensive Care Specialist Cluj‐Napoca Romania

**Keywords:** germ cell tumors, non‐seminomatous tumors, seminomatous tumors, testicular cancer

## Abstract

**Background:**

Testicular cancer represents the most common solid tumor in young adult males and is associated with excellent long‐term survival outcomes.

**Aim:**

The aim of this study is to evaluate clinical and pathological characteristics, treatment strategies, and survival outcomes of a large Romanian institutional cohort.

**Methods and Results:**

We conducted a retrospective study including 246 patients with testicular cancer treated at a tertiary oncology center between January 2005 and December 2015. Clinical, pathological, and treatment data were extracted from institutional medical records, and survival outcomes were obtained from administrative registries. Overall survival was estimated using the Kaplan–Meier method and compared between groups using the log‐rank test. Cox proportional hazards regression was used to estimate adjusted hazard ratios with 95% confidence intervals. The study included 246 patients, with a mean age at diagnosis of 32.3 years. Non‐seminomatous mixed germ cell tumors were the most frequent histological subtype, followed by seminoma. Stage I disease was the most common presentation, while approximately one‐third of patients had advanced disease. At last follow‐up, 210 patients (85.4%) were alive. Survival was significantly better in early‐stage disease and worse among patients with advanced stage and poor IGCCCG risk.

**Conclusion:**

In this Romanian cohort, nearly one‐third of patients with testicular cancer presented with advanced‐stage disease, and non‐seminomatous tumors were the most frequent histological type. Long‐term outcomes were excellent for stage I–II disease, while advanced stage and poor IGCCCG risk were associated with worse survival.

## 1 Introduction

Testicular cancer accounts for approximately 1% of all malignancies in adult males, yet it represents the most common solid tumor in young men aged 15–40 years, thereby resulting in a marked impact on morbidity and long‐term outcomes in this population [1, 2]. Globally, the incidence of testicular cancer has shown a consistent increase in recent decades, with notable geographic variation, characterized by higher rates in Northern and Western Europe and lower incidence in Eastern European countries [2] In Romania, testicular cancer is considered a relatively low‐incidence malignancy, consistent with the epidemiological pattern observed across most Eastern European countries [3]. However, contemporary national clinical data on testicular cancer in Romania remain scarce. Most of the available evidence derives from retrospective single‐center studies, while national reports largely provide overall cancer incidence estimates without detailed data on treatment strategies or clinical outcomes [4]. This likely reflects the absence of a well‐established national cancer registry and the limited availability of standardized outcome reporting, which limits the development of strong national‐level evidence and the accurate evaluation of treatment patterns and outcomes [5, 6, 7].

Germ cell tumors account for nearly 95% of all testicular malignancies, and are broadly classified into seminomatous and non‐seminomatous subtypes. Compared with seminomas, non‐seminomatous germ cell tumors are associated with a less favorable prognosis and frequently exhibit mixed histological patterns: embryonal carcinoma, yolk sac tumor, choriocarcinoma or immature teratoma [8]. Radical orchiectomy is the primary therapeutic intervention and is also mandatory for diagnosis. Pathological features, tumor markers elevations (α‐feto protein, β‐human chorionic gonadotropin, lactate dehydrogenase) and the assessment of distant metastases determine further treatment approach: active surveillance, lymphadenectomy or radiotherapy of the para‐aortic and ipsilateral iliac lymph nodes or chemotherapy [9]. Following the introduction of cisplatin‐based chemotherapy along with the implementation of multidisciplinary care, survival outcomes have markedly improved, but treatment‐related toxicities, long‐term complications, and the risk of late relapse remain clinically significant [10, 11]. Moreover, outcomes may vary across regions due to multiple factors, including disparities in healthcare access, stage at presentation, and differences in treatment approaches. These aspects are of particular importance considering the young age of affected patients and their long life expectancy.

Data from Eastern Europe, and particularly from Romania, remain scarce, limiting an extensive understanding of long‐term outcomes and regional clinical, pathological, and epidemiological features. Therefore, the aim of this study is to provide a comprehensive evaluation of patients diagnosed with testicular cancer in a Romanian cohort, focusing on clinical features, histological subtypes, stage distribution at diagnosis, treatment approaches, and survival outcomes. By addressing existing gaps in regional data, this study seeks to contribute to a better understanding of disease patterns and to support the development of more tailored, evidence‐based management strategies.

## 2 Materials and Methods

### 2.1 Study Design

This single‐center retrospective observational cohort study was conducted at the Oncology Institute “Prof. Dr. Ion Chiricuță” in Cluj‐Napoca, a tertiary referral oncology center in Romania, and included 246 patients diagnosed with testicular germ cell tumors between January 2005 and December 2015. The study period was selected to ensure adequate long‐term follow‐up, allowing for the reliable estimation of 10‐ and 15‐year overall survival outcomes. In addition, restricting the cohort to this timeframe ensured a relatively homogeneous treatment era and minimized potential bias related to temporal changes in diagnostic pathways and therapeutic strategies.

### 2.2 Study Population

#### 2.2.1 Inclusion Criteria

- Patients diagnosed with testicular germ cell tumors at the Oncology Institute “Prof. Dr. Ion Chiricuță” in Cluj‐Napoca during the study period between January 2005 and December 2015
- Histologically confirmed testicular germ cell tumors.
- Availability of baseline clinicopathological and treatment data.
- Availability of follow‐up and survival outcome data

#### 2.2.2 Exclusion Criteria

- Non‐germ cell testicular malignancies
- Patients with incomplete or missing essential clinical data
- Lack of histopathological confirmation
- Patients lost to follow‐up immediately after diagnosis or with insufficient follow‐up information
- Patients treated exclusively outside the study institution without accessible treatment records

### 2.3 Data Collection

Demographic, clinical, pathological, and treatment‐related variables were extracted from electronic medical records and institutional databases at the Oncology Institute “Prof. Dr. Ion Chiricuță” in Cluj‐Napoca. Collected variables included age at diagnosis, histological subtype, clinical stage, International Germ Cell Cancer Collaborative Group (IGCCCG) risk classification, serum tumor markers, treatment‐related variables considered relevant to prognosis, and survival outcomes.

Tumors were classified according to the World Health Organization (WHO) histopathological classification and staged using the American Joint Committee on Cancer (AJCC) TNM staging system valid at the time of diagnosis.

### 2.4 Therapeutic Approach

All patients were managed according to institutional protocols, with radical inguinal orchiectomy performed as the standard diagnostic and therapeutic initial procedure.

During the study period (2005–2015), most patients with stage I disease received adjuvant chemotherapy, while active surveillance was less frequently used, reflecting the prevailing practice at that time. Stage I seminoma was primarily treated with one or two cycles of carboplatin (AUC 7), whereas stage I non‐seminomatous germ cell tumors (NSGCTs) most commonly received two cycles of BEP chemotherapy.

Patients with stage II–III disease were treated with cisplatin‐based combination chemotherapy, most commonly BEP for three to four cycles, with EP used in cases where bleomycin was contraindicated or discontinued due to toxicity. In the salvage setting, VeIP and TIP were the most frequently used regimens, with additional gemcitabine‐, taxane‐, or platinum‐based combinations applied in later lines depending on prior therapy and clinical status.

Surgical resection of residual masses, mainly retroperitoneal lymph node dissection, was rarely required, and no patients received radiotherapy during the study period.

Additional treatment details are provided in Supporting Information S1.

### 2.5 Study Endpoints

The primary endpoint was overall survival (OS), evaluated at 5, 10, and 15 years. OS was defined as the time from diagnosis to death from any cause. For patients who were still alive at the last available follow‐up, survival time was calculated up to the date of their last recorded follow‐up.

Secondary endpoints included the assessment of clinicopathological and treatment‐related factors associated with OS. Survival was estimated using the Kaplan–Meier method and compared between groups using the log‐rank test. Cox proportional hazards regression was used to estimate hazard ratios with 95% confidence intervals.

### 2.6 Statistical Analysis

Baseline demographic, clinical, and pathological characteristics were summarized using descriptive statistics. Categorical variables were reported as frequencies and percentages, while continuous variables were summarized as medians with ranges or means with standard deviations as appropriate.

Overall survival (OS) was estimated using the Kaplan–Meier method. Survival curves were compared between groups using the log‐rank test. Cox proportional hazards regression was used to estimate hazard ratios (HRs) with 95% confidence intervals (CIs) and *p*‐values for the groups shown in the survival curves. Multivariable models were adjusted for age at diagnosis and available clinicopathological factors, as appropriate.

Because disease stage and IGCCCG prognostic group are clinically related, separate Cox models were used to avoid overadjustment and collinearity. Stage‐specific models were adjusted for age at diagnosis and histology. IGCCCG‐specific models were adjusted for age at diagnosis and histology. For histology, two sensitivity models were fitted: one adjusted for age at diagnosis and disease stage, and one adjusted for age at diagnosis and IGCCCG prognostic group. For treatment‐group analyses, adjusted survival curves were generated from a multivariable Cox proportional hazards model adjusted for age at diagnosis, disease stage, histology, and IGCCCG prognostic group.

The proportional hazards assumption was assessed using Schoenfeld residuals. A two‐sided *p* < 0.05 was considered statistically significant. All statistical analyses were performed using R software (version 4.x; R Foundation for Statistical Computing, Vienna, Austria), including the survival and survminer packages.

## 3 Results

### 3.1 Clinicopathological Characteristics

**FIGURE 1:**
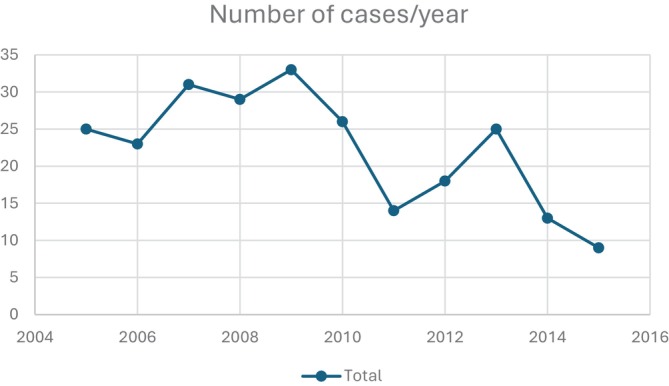
Annual incidence of newly diagnosed testicular cancer cases.

**FIGURE 2:**
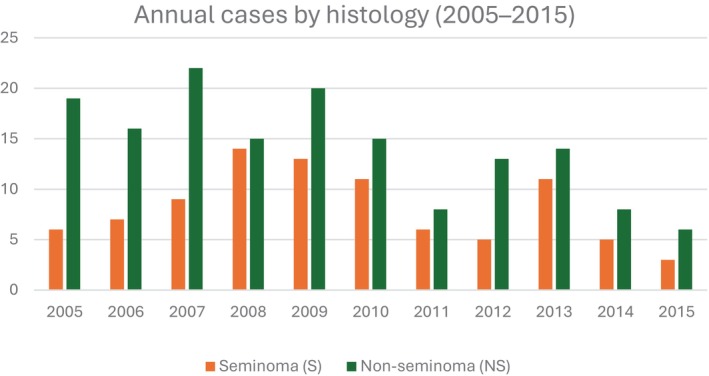
Annual number of newly diagnosed testicular cancer cases by histological subtype.

Figure 1 presents the annual number of newly diagnosed cases, while Figure 2 provides a stratified analysis according to histological subtype, emphasizing temporal trends and changes in disease distribution throughout the study period from January 2005 to December 2015. Each histological category is displayed individually, enabling direct comparison of incidence patterns and their evolution over time.

The overall average age at diagnosis was 32.3 years, with patients ranging in age from 15 to 71 years. When analyzed by histological subtype, individuals with non‐seminomatous tumors were diagnosed at a younger average age of 29.8 years (range 15–61), whereas patients with seminoma had a higher average age at diagnosis of 36.6 years (range 20–71).

Table 1 summarizes the distribution of cases according to histological subtype, providing an overview of the relative frequencies of seminomatous and non‐seminomatous tumors within the study cohort. Among the 246 patients diagnosed with germ cell testicular cancer, 90 cases (36.58%) were classified as seminoma, while the majority, 156 patients (63.41%), presented non‐seminomatous tumors. Within the seminoma subgroup, only three cases corresponded to the spermatocytic variant, whereas the remaining cases were consistent with the classic seminoma histology. Within the non‐seminomatous group, a large proportion of cases consisted of mixed germ cell tumors, defined by the presence of more than two distinct histological components, accounting for 45.12% of cases. In terms of individual histological subtypes, embryonal carcinoma represented 13% of cases, followed by yolk sac tumors (2.84%), choriocarcinoma (1.62%), and teratoma (0.81%).

**TABLE 1:** Distribution of histological subtypes.

Histological subtypes	No. of cases 246	Percentage 100%
Seminoma	90	**36.58%**
Classic	87	35.36%
Spermatocytic	3	1.21%
Non‐seminoma	156	**63.41%**
Mixed tumors	111	45.12%
Embrional carcinoma	32	13.00%
Yolk sac	7	2.84%
Choriocarcinoma	4	1.62%
Teratoma	2	0.81%

*Note:* The bold values represent the main subtypes of testicular cancer, which are further classified into distinct histological subgroups.

Table 2 describes Clinical Stage and Metastatic Site Distribution Stratified by Histological Subtype. Staging was conducted according to the American Joint Committee on Cancer (AJCC) TNM + S staging system, which included tumor size and extent (T), regional lymph node involvement (N), the presence of distant metastases (M), and S (serum tumor markers). Among the 246 patients included in the analysis, Stage I disease was the most frequent, comprising 112 patients (45.5%), followed by Stage III disease in 81 patients (32.9%) and Stage II in 53 patients (21.5%). When stratified by histology, seminomas were most frequently diagnosed at Stage I with 55 cases (22.36%), whereas patients presenting with non‐seminomas had advanced disease more frequently. Stage III disease was markedly higher among non‐seminoma patients, representing 66 cases (26.83%), compared with only 15 cases (6.10%) among seminoma patients. Similarly, Stage II disease was slightly more frequent in the non‐seminoma group (33 cases; 13.41%) compared with the seminoma group (20 cases; 8.13%). Stage IS showed a notable predominance in non‐seminoma cases (29 patients; 11.79%) compared with seminoma (5 patients; 2.03%). Regarding metastatic distribution, pulmonary or non‐retroperitoneal nodal metastases were relatively uncommon, occurring in 14 patients (5.69%) overall, including 4 cases (1.63%) of seminoma and 10 cases (4.07%) of non‐seminoma. In contrast, other visceral metastases or unspecified metastatic sites were observed in 67 patients (27.24%). The majority of these occurred in the non‐seminoma subgroup, accounting for 56 cases (22.76%), whereas only 11 cases (4.47%) were identified among seminoma patients.

**TABLE 2:** Clinical stage and metastatic site distribution stratified by histological subtype.

Stages	Seminoma	Non‐seminoma	Total
I	50 (20.33%)	28 (11.38%)	78 (31.7%)
IS	5 (2.03%)	29 (11.79%)	34 (13.82%)
II	20 (8.13%)	33 (13.41%)	53 (21.54%)
III	15 (6.10%)	66 (26.83%)	81 (32.93%)
*Pulmonary/non‐retroperitoneal nodal metastases*	4 (1.63%)	10 (4.07%)	14 (5.69%)
*Other visceral metastases/not specified*	11 (4.47%)	56 (22.76%)	67 (27.24%)

Table 3 shows the distribution of serum tumor marker categories (S‐stage) according to histological subtype. Among patients with seminoma, the majority presented with normal marker levels (S0), accounting for 49 cases, followed by S1 with 15 cases. Elevated marker levels were less frequent in this group, with 11 cases classified as S2 and only 4 cases as S3. Additionally, 11 patients were categorized as Sx, indicating unavailable or unassessed marker levels. In contrast, patients with non‐seminoma demonstrated a higher frequency of elevated serum tumor markers. The most common category in this group was S1, comprising 61 cases, followed by S0 with 34 cases. Higher marker levels were also more prevalent in non‐seminoma, with 30 cases classified as S2 and 19 cases as S3. A smaller number of patients (12 cases) were recorded as SX.

**TABLE 3:** Distribution of serum tumor marker levels (S—stage) according to histological subtype.

S—Serum markers	Seminoma	Non‐seminoma
Sx	11	12
S0	49	34
S1	15	61
S2	11	30
S3	4	19

Table 4 displays the distribution of patients according to the International Germ Cell Cancer Collaborative Group (IGCCCG) prognostic classification, stratified by histological subtype. A substantial proportion of patients in both groups was subgrouped in the good prognosis category, comprising 77 seminoma cases and 79 non‐seminoma cases. In the intermediate prognosis group, a notable difference was observed between the two subtypes, with only 5 seminoma patients compared to 37 patients in the non‐seminoma group. Additionally, a small number of cases were not specified, including 8 seminoma and 5 non‐seminoma patients.

**TABLE 4:** Distribution of patients stratified according to IGCCCG.

	Seminoma	Non‐seminoma	Total
Good prognosis	77 (31.3%)	79 (32.1%)	156 (63.4%)
Intermediate prognosis	5 (2%)	37 (15%)	42 (17.1%)
Poor prognosis	NA	35 (14.2%)	35 (14.2%)
Not specified	8 (3.3%)	5 (2%)	13 (5.3%)

### 3.2 Treatment and Therapeutic Results

Table 5 summarizes the treatment modalities administered according to histology and stage. Among seminoma patients (*n* = 90), Stage I disease (*n* = 55) was most frequently managed with carboplatin (44 patients), while surveillance alone was applied in 3 cases. Stage II–III seminoma patients (*n* = 35) predominantly received BEP chemotherapy (31 patients). In the non‐seminoma group (*n* = 156), Stage I patients (*n* = 57) were primarily treated with EP or BEP chemotherapy, with 32 patients receiving ≥ 2 cycles of BEP. Stage II–III non‐seminoma patients (*n* = 99) most frequently received BEP (84 patients), followed by EP (11 patients). In this cohort, only one patient underwent retroperitoneal lymphadenectomy to remove residual disease in the retroperitoneal nodes, and none received radiotherapy.

**TABLE 5:** Distribution of treatment strategies by histological subtype and clinical stage.

Treatment	Seminoma (90)		Nonseminoma (156)	
	Stage I (55)	Stage II—III (35)	Stage I (57)	Stage II—III (99)
Surveillance	3	0	1	0
Carboplatin	44	2	0	0
EP	3	2	22	11
BEP	3	31	34	84
Other	2	0	0	3
RTE	0	0	0	0
Lymphadenectomy	0	0	0	1

Table 6 displays the number of chemotherapy cycles administered for Stage I patients stratified by histology. In Stage I seminoma, carboplatin was most often given as a single cycle (32 patients), with fewer patients receiving ≥ 2 cycles (12 patients). In Stage I non‐seminoma, the majority of patients received at least 2 cycles of BEP (32 patients) or EP (22 patients), while very few received only 1 cycle (2 patients).

**TABLE 6:** Number of chemotherapy cycles in Stage I testicular cancer by histology.

Stage I				
Treatment	Seminoma		Non‐seminoma	
	1 cycle	2 cycles	1 cycles	≥ 2 cycles
Carboplatin	32	12	0	0
BEP	0	3	2	32
EP	0	3	0	22

### 3.3 Survival Outcomes

**FIGURE 3:**
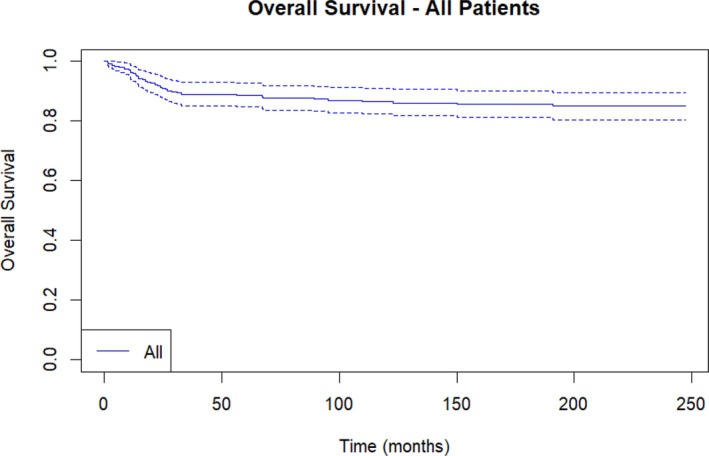
Overall survival for all patients.

In this cohort of 246 patients with testicular cancer diagnosed between 2005 and 2015, with an average follow‐up of 188 months, overall survival (OS) remained high. For the entire cohort, OS at 5, 10, and 15 years was 88.6% (95% CI, 84.7–92.7), 86.6% (95% CI, 82.4–91.0), and 85.7% (95% CI, 81.4–90.2), respectively (Figure 3, Table 7).

**TABLE 7:** Overall survival for all patients at 5, 10, 15 years.

Years	OS (%)	95% CI lower	95% CI upper
5	88.6	84.7	92.7
10	86.6	82.4	91.0
15	85.7	81.4	90.2

**FIGURE 4:**
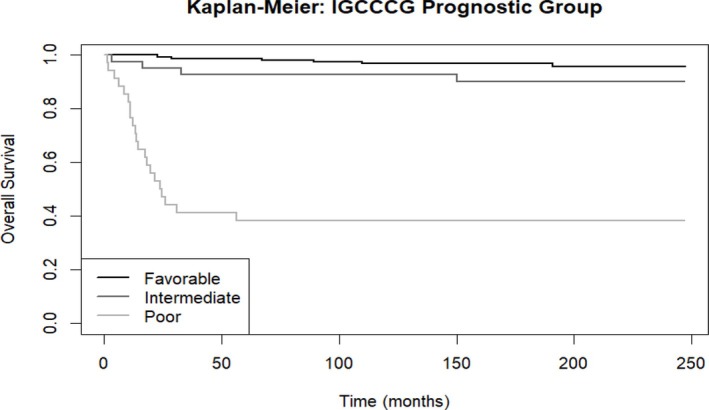
Overall survival by IGCCCG prognosis. Global log‐rank *p* < 0.001.

When stratified by IGCCCG risk group, OS differed significantly between groups by log‐rank test (*p* < 0.001). Patients in the favorable‐risk group had OS of 98.7% (95% CI, 97.0–100) at 5 years and 96.8% (95% CI, 94.1–99.6) at both 10 and 15 years. Intermediate‐risk patients had OS of 92.9% (95% CI, 85.4–100) at 5 and 10 years, decreasing to 90.2% (95% CI, 81.5–99.8) at 15 years. In contrast, the poor‐risk group had markedly lower OS, remaining 38.2% (95% CI, 24.9–58.6) across all time points (Figure 4, Table 8). In the Cox model adjusted for age at diagnosis and histology, intermediate‐risk disease (adjusted HR 5.06, 95% CI, 1.31–19.60; *p* = 0.019) and poor‐risk disease (adjusted HR 71.10, 95% CI, 17.50–289.00; *p* < 0.001) were associated with worse OS compared with favorable‐risk disease (Table 9). Given the limited number of deaths, particularly within some IGCCCG subgroups, the magnitude of the adjusted estimate for the poor‐risk group should be interpreted with caution.

**TABLE 8:** Overall survival by IGCCCG prognosis at 5, 10, 15 years.

Comparison	Adjusted HR	95% CI	Cox *p*‐value
Intermediate vs. favorable	5.06	1.31–19.60	0.019
Poor vs. favorable and IGCCCG prognostic group are clinically related.	71.10	17.50–289.00	< 0.001

**TABLE 9:** Adjusted hazard ratios for IGCCCG prognostic group.

IGCCCG risk	Years	OS (%)	95% CI lower	95% CI upper
Favorable	5	98.7	97.0	100
Favorable	10	96.8	94.1	99.6
Favorable	15	96.8	94.1	99.6
Intermediate	5	92.9	85.4	100
Intermediate	10	92.9	85.4	100
Intermediate	15	90.2	81.5	99.8
Poor	5	38.2	24.9	58.6
Poor	10	38.2	24.9	58.6
Poor	15	38.2	24.9	58.6

*Note:* Adjusted HRs are from a Cox model adjusted for age at diagnosis and histology. Stage was not included in this model because disease stage.

**FIGURE 5:**
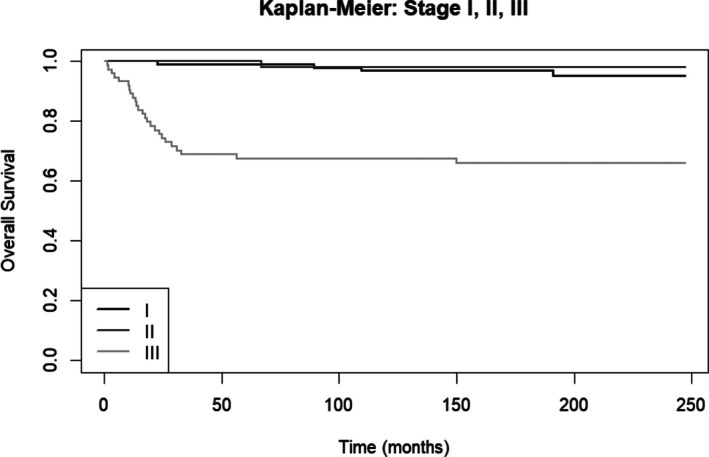
Overall survival by stage. Global log‐rank *p* < 0.001.

When stratified by clinical stage, patients with early disease had excellent outcomes, while overall survival differed significantly across groups by log‐rank testing (*p* < 0.001). Stage I patients had OS of 99.0% (95% CI, 97.0–100) at 5 years and 96.9% (95% CI, 93.5–100) at both 10 and 15 years. Stage II patients had 100% (95% CI, 100–100) OS at 5 years and 98.1% (95% CI, 94.5–100) at 10 and 15 years. Stage III patients had lower long‐term survival, with OS of 67.6% (95% CI, 57.7–79.1) at 5 and 10 years and 66.0% (95% CI, 56.0–77.8) at 15 years (Figure 5, Table 10). In the Cox model adjusted for age at diagnosis and histology (Table 11), Stage III disease was associated with worse OS compared with Stage I disease (adjusted HR 9.58, 95% CI, 3.65–25.20; *p* < 0.001), whereas Stage II was not significantly different from Stage I (adjusted HR 0.45, 95% CI, 0.05–3.83; *p* = 0.462). Stage‐specific estimates, particularly for stage II disease, should be interpreted cautiously because of the limited number of deaths in some subgroups.

**TABLE 10:** Overall survival by stage at 5, 10, 15 years.

Stage	Years	OS (%)	95% CI lower	95% CI upper
I	5	99.0	97.0	100
I	10	96.9	93.5	100
I	15	96.9	93.5	100
II	5	100	100	100
II	10	98.1	94.5	100
II	15	98.1	94.5	100
III	5	67.6	57.7	79.1
III	10	67.6	57.7	79.1
III	15	66.0	56.0	77.8

**TABLE 11:** Adjusted hazard ratios for disease stage.

Comparison	Adjusted HR	95% CI	Cox *p*‐value
Stage II vs. Stage I	0.45	0.05–3.83	0.462
Stage III vs. Stage I	9.58	3.65–25.20	< 0.001

*Note:* Adjusted HRs are from a Cox model adjusted for age at diagnosis and histology. IGCCCG prognostic group was not included in this model because disease stage and IGCCCG prognostic group are clinically related.

**FIGURE 6:**
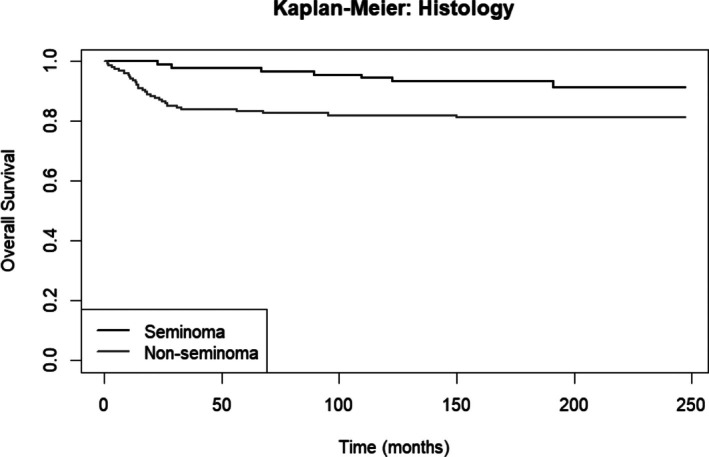
Overall survival by histology. Global log‐rank *p* = 0.017.

By histology, OS differed significantly between seminoma and non‐seminoma in the unadjusted log‐rank analysis (*p* = 0.017). OS for seminoma was 97.8% (95% CI, 94.8–100) at 5 years, 94.4% (95% CI, 89.8–99.3) at 10 years, and 93.3% (95% CI, 88.3–98.6) at 15 years. Non‐seminoma patients had OS of 83.3% (95% CI, 77.7–89.4) at 5 years, 82.1% (95% CI, 76.2–88.3) at 10 years, and 81.3% (95% CI, 75.4–87.7) at 15 years (Figure 6, Table 12). However, in sensitivity Cox models, histology was not independently associated with OS. For seminoma versus non‐seminoma, the adjusted HR was 0.48 (95% CI, 0.20–1.15; *p* = 0.098) after adjustment for age and stage, and 2.08 (95% CI, 0.53–8.20; *p* = 0.293) after adjustment for age and IGCCCG prognostic group (Table 13). Differences between the stage‐adjusted and IGCCCG‐adjusted estimates likely reflect the close relationship between histology, metastatic risk classification, and disease burden, as IGCCCG incorporates prognostic features that overlap with, but are not equivalent to, clinical stage and are defined differently for seminoma and non‐seminoma.

**TABLE 12:** Overall survival by histology at 5, 10, 15 years.

Histology	Years	OS (%)	95% CI lower	95% CI upper
Seminoma	5	97.8	94.8	100
Seminoma	10	94.4	89.8	99.3
Seminoma	15	93.3	88.3	98.6
Non‐seminoma	5	83.3	77.7	89.4
Non‐seminoma	10	82.1	76.2	88.3
Non‐seminoma	15	81.3	75.4	87.7

**TABLE 13:** Adjusted hazard ratios for histology.

Comparison	Adjusted HR	95% CI	Cox *p*‐value
Seminoma vs. non‐seminoma (adjusted for age and stage)	0.48	0.20–1.15	0.098
Seminoma vs. non‐seminoma (adjusted for age and IGCCCG)	2.08	0.53–8.20	0.293

*Note:* Two sensitivity Cox models were fitted for histology: one adjusted for age at diagnosis and disease stage, and one adjusted for age at diagnosis and IGCCCG prognostic group. Reference category: non‐seminoma.

**FIGURE 7:**
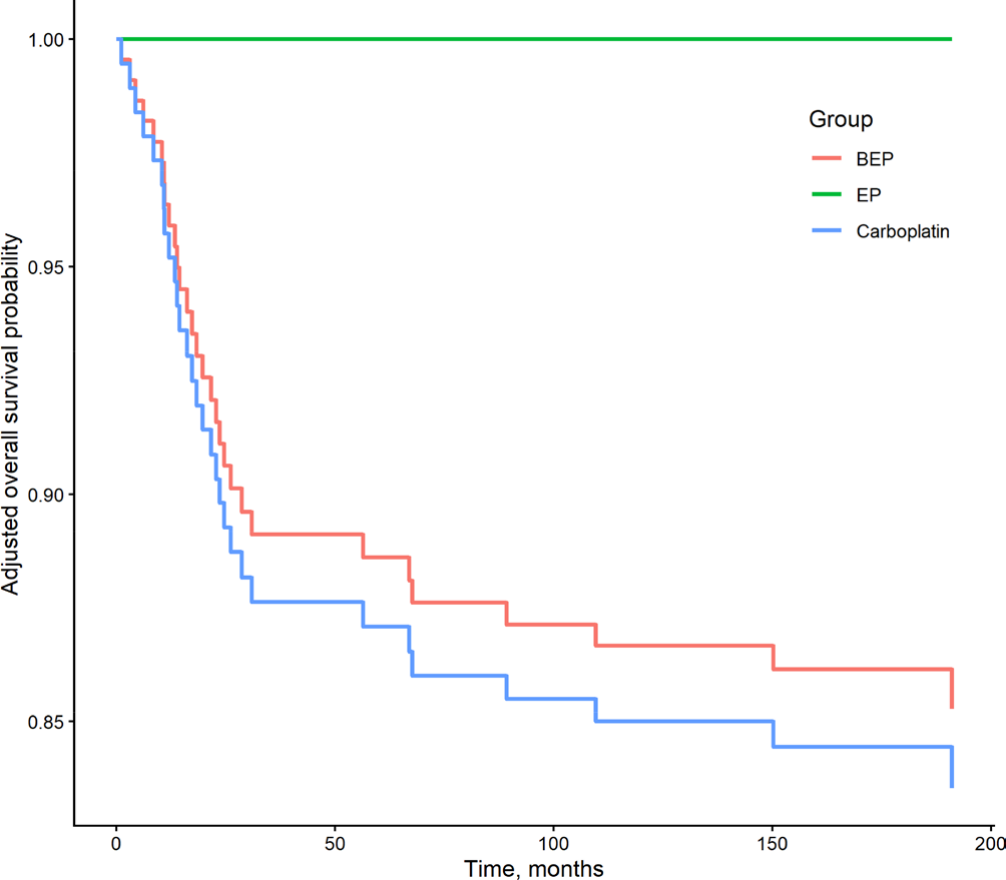
Adjusted overall survival curves by treatment group.

Adjusted survival curves were generated by treatment group for BEP, EP, and carboplatin using the multivariable Cox model adjusted for age at diagnosis, disease stage, histology, and IGCCCG prognostic group (Figure 7). The adjusted 5‐, 10‐, and 15‐year OS estimates were 88.6%, 86.7%, and 86.1% for BEP; 100%, 100%, and 100% for EP; and 87.1%, 85.0%, and 84.4% for carboplatin, respectively (Table 14). The EP estimates should be interpreted cautiously because no deaths occurred in the EP group.

**TABLE 14:** Adjusted overall survival estimates by treatment group.

Treatment group	5‐year adjusted OS	10‐year adjusted OS	15‐year adjusted OS
BEP	88.6% (83.9–93.3)	86.7% (81.5–91.8)	86.1% (80.8–91.5)
EP	100% (100–100)	100% (100–100)	100% (100–100)
Carboplatin	87.1% (71.4–100)	85.0% (68.1–100)	84.4% (67.2–100)

## 4 Discussion

Testicular cancer remains a relatively uncommon malignancy in Romania, with national epidemiological data suggesting a comparatively low disease burden and a limited number of reported cases annually [12]. In this study, which included 246 patients diagnosed with testicular cancer at a tertiary oncology center during the period between January 2005–December 2015, the incidence per year ranged from 9 to 33 cases, with a mean of 22–23 cases per year. Institutional case series from other tertiary centers demonstrate a wide range of annual caseloads for testicular cancer; for instance, data from a UK referral center reported approximately 50 cases per year over a 12‐year period [13], while a tertiary cancer center in Northeast India registered about 14–15 cases annually [14]. This variation reflects underlying differences in regional incidence, referral patterns, and the distribution of risk factors.

The study demonstrated a decreasing trend in the annual number of testicular cancer cases within our institutional cohort, contrasting with the increasing incidence rates reported in population‐based studies from Western Europe and North America [2]. This discrepancy may be explained by the fact that our data derive from a single tertiary oncology referral center and therefore reflect institutional case volume rather than true population‐based incidence. Temporal changes in referral patterns, increasing decentralization of oncological care, and management of selected early‐stage cases outside our institution may also have influenced these findings. Consequently, the decline identified in our cohort likely reflects institutional dynamics and healthcare system reorganization rather than a true reduction in disease incidence at the population level.

Clinical characteristics, including the mean age at diagnosis, were consistent with previously published series, with non‐seminomatous tumors presenting at an average age of 29.8 years and seminomas at 36.6 years [14, 15], highlighting the different age distribution between these two histologic subtypes.

In our cohort, non‐seminomatous tumors predominated (63.4%) compared to seminomas (36.6%), a pattern that differs from population‐based registries in Italy, Germany, and the USA [15, 16, 17]. In contrast, our findings are consistent with reports from similar cohorts in India and Turkey [14, 18]. These differences are most likely explained by healthcare system–related factors rather than underlying biological differences. Non‐seminomatous tumors are generally associated with a more aggressive clinical course and tend to present at a younger age and at more advanced stages, which may contribute to their overrepresentation in tertiary referral centers, while early‐stage seminomas are more likely to be managed in peripheral institutions. From a biological perspective, testicular germ cell tumors arise from a complex interaction between genetic susceptibility and early developmental factors, including cryptorchidism, impaired spermatogenesis, and features of testicular dysgenesis syndrome. However, no population‐specific biological determinants explaining differences in histological subtype distribution have been clearly established [19, 20].

Clinical stage distribution reports show seminoma is more frequently diagnosed at earlier clinical stages, with a Stage I to Stage II–III ratio of 55:35 (≈1.6:1), whereas in non‐seminoma advanced stages predominated, with a Stage I to Stage II–III ratio of 57:99 (≈0.6:1) and a higher prevalence of visceral metastases. Our cohort demonstrates a higher proportion of patients presenting with Stage II–III disease, particularly among non‐seminomas, compared to published population‐based series [17, 18]. This finding may partly reflect the tertiary referral profile of our center, which is more likely to receive complex and advanced cases, whereas some early‐stage patients may be managed in peripheral institutions. At the same time, broader healthcare‐system factors in Romania, including variable access to specialist care, delayed referral pathways, and limited awareness of testicular cancer symptoms among young men, may contribute to later‐stage presentation. This observation is consistent with prior reports indicating that education regarding testicular cancer remains suboptimal in adolescents and young adults, and that only a minority of pediatricians routinely provide structured counseling on this topic [21]. Lack of early preventive education may contribute to delayed presentation and more advanced stage at diagnosis, highlighting the need for improved awareness strategies and incorporation of testicular health education into adolescent and primary care practice.

Consistent with TNM + S staging, our findings indicate that elevated serum tumor markers are more frequently observed in non‐seminomatous tumors compared with seminomas. This distribution suggests the typically higher tumor burden and increased biological activity of non‐seminomatous germ cell tumors, which may have implications for risk stratification and treatment planning.

According to IGCCCG classification, 63.4% of patients in our cohort had a good prognosis, 17.1% an intermediate prognosis, and 14.2% a poor prognosis, closely mirroring the distribution reported in the IGCCCG consensus classification [22].

In this study, adjuvant chemotherapy was administered to most patients with Stage I disease. It is important to note that during the period 2005–2015, the prognostic significance and clinical applicability of these factors were under continuous refinement, and risk‐adapted strategies had not yet been fully established or routinely implemented in clinical practice. An illustrative example of this approach can be found in earlier studies of stage I testicular cancer. In seminoma, the landmark MRC TE19/EORTC 30982 trial, published in 2005, established single‐agent carboplatin as an effective adjuvant option in an unselected patient population, without including established risk factors such as tumor size or rete testis invasion [23]. Similarly, in non‐seminomatous germ cell tumors, early clinical series employed adjuvant treatment across all stage I patients, irrespective of lymphovascular invasion status [24]. This approach was later supported and confirmed by the SWENOTECA trial in which adjuvant BEP substantially reduced relapse in both patients with lymphovascular invasion (LVI+) (3.2%) and without LVI (LVI–) (1.3%) [25]. These non–risk‐adapted approaches resulted in low relapse rates but raised concerns regarding overtreatment, ultimately leading to the development of contemporary risk‐adapted strategies based on prognostic factors [26, 27].

In advanced‐stage disease (Stage II–III), patients received three to four cycles of BEP, while EP was used in those unable to tolerate bleomycin. When bleomycin was discontinued due to cumulative dose limits or toxicity—most commonly pulmonary—the remaining cycles were completed with etoposide and cisplatin (EP).

With an average follow‐up of 188.7 months, the overall survival (OS) for the entire cohort was 88.6% at 5 years and remained consistent later in time. The slightly lower survival rates compared to other population‐based series [28, 29] are likely due to the high proportion of patients with Stage III disease and non‐seminomatous histology in our cohort. On the other hand, patients with Stage I and II disease achieved excellent overall survival that was near or at 100% throughout follow‐up. The remarkable OS observed in stage I patients aligns with findings from other trials, where the administration of adjuvant chemotherapy irrespective of risk status provided excellent survival outcomes [25].

The additional adjusted analyses reinforce the central role of disease burden and prognostic risk in long‐term outcomes, consistent with the original IGCCCG classification and its modern update [22, 30]. The poorer survival observed among stage III patients remained evident after accounting for age and histology, suggesting that advanced stage at presentation is a major driver of mortality in this cohort. Similarly, IGCCCG risk stratification retained strong prognostic value, with poor‐risk disease identifying a subgroup with particularly unfavorable long‐term survival. Although the effect estimate for poor IGCCCG risk was large, the wide confidence interval reflects the limited number of events and highlights the need for cautious interpretation.

Patients in the favorable and intermediate prognostic groups demonstrated improved 5‐year survival compared with the one reported in the IGCCCG consensus classification [22, 30]. In contrast, patients with stage III disease and those in the unfavorable IGCCCG prognostic group had the poorest outcomes, with 5‐year overall survival of 67.6% and 38.2%, respectively, which are lower than reported in other series [29].

Overall, these findings support the clinical relevance of early diagnosis and risk‐adapted management in improving outcomes for patients with testicular cancer.

Although seminoma patients showed better OS than non‐seminoma patients in the unadjusted Kaplan–Meier analysis, histology was not independently associated with OS in sensitivity Cox models adjusted for either age and stage or age and IGCCCG prognostic group. This suggests that the apparent survival difference by histology may be partly explained by differences in stage distribution and prognostic risk rather than histology alone, consistent with established risk‐adapted prognostic models in testicular germ cell tumors [22, 30].

Regarding treatment groups, adjusted survival estimates were favorable among patients receiving BEP, EP, or carboplatin; however, comparisons between regimens should be interpreted cautiously because treatment allocation was non‐randomized and likely influenced by disease stage, histology, IGCCCG risk group, comorbidities, and clinician preference. The 100% estimated survival observed in the EP group likely reflects the absence of deaths in this subgroup and possible selection of lower‐risk patients, rather than evidence of superiority over BEP or carboplatin.

### 4.1 Strengths and Limitations

This study has several strengths. It represents one of the few single‐institution Eastern European cohorts reporting long‐term outcomes in testicular germ cell tumors, providing valuable real‐world data from this region. The relatively large cohort and extended follow‐up period allowed robust estimation of long‐term survival outcomes, including 10‐ and 15‐year OS. In addition, the homogeneous treatment era (2005–2015) improved internal consistency by minimizing variability related to changes in diagnostic and therapeutic approaches over time.

Several limitations should be acknowledged. First, the retrospective design is associated with potential selection bias and missing data, which may affect the robustness of some findings. Second, the single‐center nature of the study at a tertiary referral institution may limit generalizability due to referral bias and potential overrepresentation of advanced or complex cases. Third, the study period (2005–2015), chosen to ensure adequate long‐term follow‐up, may reduce applicability to current treatment practices. Fourth, age at diagnosis was the only demographic variable available in the structured dataset, limiting the ability to adjust for other potential demographic or socioeconomic factors. Finally, treatment‐specific comparisons should be interpreted cautiously because treatment allocation was not randomized and was influenced by disease stage and prognostic risk; in addition, no deaths occurred in the EP group, limiting estimation and interpretation for this subgroup.

## 5 Conclusion

In conclusion, this study shows that the clinical pattern of testicular cancer in this Romanian cohort is broadly comparable to that reported in other regions, although a higher proportion of non‐seminomatous tumors and advanced‐stage disease were observed. With extended long‐term follow‐up, our analysis provides detailed survival estimates by disease stage, IGCCCG prognostic group, histology, and treatment group. Advanced stage and poor IGCCCG risk were the main factors associated with worse survival, underscoring the importance of early diagnosis, timely referral, and risk‐adapted management in this population.

## Author Contributions

**Ciprian Deac:** writing – review and editing, conceptualization. **Emanuiela Florentina Rohozneanu:** conceptualization, investigation, data curation. **Călin Ioan Căinap:** supervision, data curation.

## Funding

Publication of this paper was supported by the “Iuliu Hațieganu” University of Medicine and Pharmacy Cluj‐Napoca through its institutional Open Access Program.

## Ethics Statement

This study was performed in accordance with the principles of the Declaration of Helsinki. Approval was granted by the Ethics Committee of The Oncology Institute “Prof. Dr. Ion Chiricută” Cluj‐Napoca (Date 12/13/2022 / No. 12123). Due to the retrospective nature of the study and the use of anonymized data, the requirement for written informed consent was waived by the Ethics Committee.

## Conflicts of Interest

The authors declare no conflicts of interest.

## Supporting information


**Data S1:** Detailed treatment strategies according to disease stage and histological subtype.

## Data Availability

The data that support the findings of this study are available from the corresponding author upon reasonable request.
