# Supplementary material for: Clinicopathological Features, Treatment Patterns, and Long‐Term Survival in Patients With Testicular Cancer: A Retrospective Cohort Study
Source: Cancer Rep (Hoboken). 2026 Jun 14;9(6):e70606. doi: 10.1002/cnr2.70606 (PMC13265246; doi:10.1002/cnr2.70606)
Supplement: Supplementary file 1 — Data S1: Detailed treatment strategies according to disease stage and histological subtype. [file CNR2-9-e70606-s001.docx]

**Supplementary File S1. Detailed treatment strategies according to disease stage and histological subtype.**

This supplementary file summarizes the treatment approaches administered to patients included in the study, stratified by disease stage and histological subtype.

All patients were managed in accordance with the institutional protocol, which served as the primary guide for treatment decisions. In cases with suspected testicular neoplasm, the first step consisted of a radical inguinal orchiectomy, performed for both diagnosis and therapeutic management.

During the study period (2005–2015), the majority of patients with stage I disease received adjuvant chemotherapy according to institutional protocols, irrespective of histopathological risk factors. Active surveillance was less commonly employed at the time, reflecting the prevailing clinical approach before risk-adapted strategies were widely adopted. This approach is consistent with current practices in numerous centers during that time frame, when adjuvant treatment was preferred for reducing relapse risk in all stage I patients.

For stage I seminoma, adjuvant chemotherapy consisting of one or two cycles was generally considered the preferred approach, with single-agent carboplatin (AUC 7) administered in most cases. Stage I non-seminomatous germ cell tumors (NSGCTs) were primarily managed with adjuvant chemotherapy, most commonly consisting of two cycles of the BEP regimen. BEP is administered in 21-day cycles and includes bleomycin, etoposide, and cisplatin. Cisplatin was given at a dose of 20 mg/m² intravenously on days 1–5, and etoposide at 100 mg/m² intravenously on days 1–5. Bleomycin was administered at a dose of 30 units intravenously on days 1, 8, and 15 of each cycle.

For patients with advanced disease (stage II–III), cisplatin-based combination chemotherapy represented the standard treatment approach. Most patients received three to four cycles of the BEP regimen, while the EP regimen was used in patients with contraindications to bleomycin.In patients treated with BEP who reached the planned cumulative dose of bleomycin or in whom bleomycin had to be discontinued because of toxicity—most commonly pulmonary toxicity—treatment was continued with etoposide and cisplatin (EP) in order to complete the proposed number of cycles. In this setting, EP was administered every 21 days, with etoposide 100 mg/m² and cisplatin 20 mg/m² given intravenously on days 1–5. This schedule maintains the same dosing intensity as the BEP regimen while avoiding further exposure to bleomycin. This strategy preserves the curative potential of platinum-based therapy while reducing the risk of bleomycin-related adverse effects, particularly interstitial pneumonitis and pulmonary fibrosis, and is widely accepted in the management of germ cell tumors when continuation of bleomycin is contraindicated.

In the second-line setting, the most frequently administered regimen was VeIP chemotherapy, followed by TIP. The VeIP regimen was administered in 21-day cycles and consisted of vinblastine 0.11 mg/kg intravenously on days 1–2, ifosfamide 1.2 g/m² intravenously on days 1–5 together with mesna for uroprotection, and cisplatin 20 mg/m² intravenously on days 1–5. The TIP regimen (paclitaxel, ifosfamide, and cisplatin) represented the second most frequently employed salvage protocol. It was administered every 21 days and included paclitaxel 250 mg/m² given as a 24-hour intravenous infusion on day 1, followed by ifosfamide 1.5 g/m² intravenously on days 2–5 with mesna added for support, and cisplatin 25 mg/m² intravenously on days 2–5, typically with granulocyte colony-stimulating factor (G-CSF) support.

In later treatment lines, several alternative regimens were used depending on prior therapy, patient performance status, and disease characteristics. These included gemcitabine-based combinations, such as gemcitabine 1000 mg/m² intravenously on days 1 and 8 combined with carboplatin (AUC 4–5 on day 1), oxaliplatin 130 mg/m² on day 1, or docetaxel 75 mg/m² on day 1, administered in 21-day cycles. Other salvage approaches included CISCA-based regimens, consisting of Cisplatin, Cyclophosphamide, and Doxorubicin, typically administered as cisplatin 20 mg/m²/day intravenously on days 1–5, cyclophosphamide 600–1000 mg/m² intravenously on day 1, and doxorubicin 50 mg/m² intravenously on day 1, with cycles repeated every 21 days. Alternatively, a combination of Paclitaxel and Carboplatin was used, typically administered as paclitaxel 175 mg/m² intravenously on day 1 together with carboplatin (AUC 5) on day 1, every 21 days.

In patients with bone marrow metastatic burden, a regimen consisting of bleomycin, cisplatin, and dexamethasone (BCP) was used according to the institutional protocol. This regimen generally included bleomycin 30 units intravenously on days 1, 8, and 15, cisplatin 20 mg/m² intravenously on days 1–5, and dexamethasone administered in high doses during the initial days of the cycle, with cycles repeated every 21 days depending on hematologic recovery and treatment tolerance.

Residual masses after chemotherapy were uncommon in this cohort. When present, the standard approach during the 2005–2015 period was surgical resection—typically retroperitoneal lymph node dissection (RPLND) for non-seminomatous germ cell tumors, or resection for seminoma if the mass was ≥3 cm and PET findings were inconclusive—aiming to remove any viable tumor or teratoma while avoiding unnecessary surgery in cases of necrosis or fibrosis. In this cohort, only one patient underwent RPLND, and no patients received radiotherapy.
